# Visual analysis of research progress and trend on hairy roots

**DOI:** 10.3389/fpls.2025.1580007

**Published:** 2025-04-29

**Authors:** Yu-Ping Zheng

**Affiliations:** Library of Henan University of Science and Technology, Henan University of Science and Technology, Luoyang, China

**Keywords:** hairy root, bibliometrics, CiteSpace, VOSvivewer, visual analysis

## Abstract

Hairy root is a special form of root tissue, which is induced by *Rhizobium rhizogenes* and could mediate genetic transformation after the infection of explants. They have attracted attentions because of their advantages of fast growth rate, convenient culture, stable genetic properties and strong synthesis ability of secondary metabolites. With bibliometrics, this study employed CiteSpace and VOSvivewer softwares to analyze the publications on hairy roots researches from 2009 to 2024 based on WOS database. The subject distribution, countries, institutions and personnel, research hotspots and research trends of hairy roots were discussed and analyzed. The results revealed a consistent increase in publications on hairy root with America, China and India as the main countries. The institutions were mainly universities and the Chinese Academy of Sciences was a major contributor to this topic and had close cooperation with other institutions. The researches of hairy roots mainly focused on plant genetic transformation, secondary metabolism and gene molecular function analysis, and in environmental remediation. The application of hairy roots could be an important research hotspot in the future.

## 1 Introduction

With the global population growth and the increasingly complex climate situation (Raihan, 2023), the contradiction between supply and demand of resources has become increasingly prominent, including food, medicine and industrial materials (Dwivedi et al., 2013; Löfgren et al., 2024). Synthetic biology is an effective way to solve scientific and social problems in agriculture and industry, and hairy roots are one of the major biosynthetic tools (Morey and Peebles, 2022; Ye et al., 2023). Hairy root, as a plant pathological phenomenon, is a special form of root tissues mediated by *Rhizobium rhizogenes* infection of plant wounds (Hu. and Du., 2006). With the characteristics of genetic stability, strong metabolic synthesis ability, convenient culture and short production cycle, plant hairy roots will be developed into an important tool for plant secondary metabolism and industrial biosynthesis (Hu. and Du., 2006; Srivastava and Srivastava, 2007; Gutierrez-Valdes et al., 2020),. Among them, secondary metabolites are considered to be special metabolites, which have a wide range of roles in agriculture, food and medicine (Dixon and Dickinson, 2024). In addition, according to these excellent properties, the application and potential of hairy root in plant function verification and environmental governance will be a research hotspot (Kiryushkin et al., 2021).

As an organized culture system, hairy roots are induced by *Rhizobium rhizogenes* with integrating T-DNA from Ri plasmid into plant genome for transcription and expression (Guillon et al., 2006; Sykłowska-Baranek et al., 2022). The induction of hairy roots is regulated by multiple factors, including plant species, explant and strain type, transformation method (bacterial concentration and infection time), medium type (rate of plant hormones or chemical inducers) and culture conditions (temperature, photoperiod and pH) (Ferguson et al., 2023; Zhu et al., 2024). Thus, there are many factors influencing hairy root induction, leading to the independence of various plant genetic transformation systems (Ron et al., 2014; Cheng et al., 2021). Hairy root has important applications in plant transformation and synthesis of secondary metabolite, however, there are not systematic analysis and summary of the present research situation about this.

Compared with a literature review, bibliometrics combines mathematical and statistical methods to analyze the quantitative relationship and development regularity of scientific literature, and then summarizes research progress and trends in related research fields (Ninkov et al., 2022; Salinas-Ríos, 2022). The development of bibliometrics benefits from the introduction of software such as Citespace and VOSviewer, which contribute to the analysis of countries, institutions, journals, authors and keywords through network analysis and clustering algorithms (Chen, 2006; Van Eck and Waltman, 2010; Mokhnacheva and Tsvetkova, 2020). The mining and analysis of keywords and co-author information in scientific literature can quickly understand the development, trends and frontier hotspots of this research field, which lays a foundation for the selection of future topics and the cooperation between different institutions (Chen, 2012; Chen et al., 2024). To date, researchers in various disciplines have established several relevant bibliometric analysis; for example, in the field of plant research, the research status and development trend of *Impatiens balsamina* and *Phalaenopsis* were described in detail (Guo WenJiao et al., 2017; Chen et al., 2024). In addition, some scholars had summarized the research status and global trends of Slow/Controlled-Release Fertilizers and Soil Carbon Sequestration (El Allaoui et al., 2024; Francis et al., 2024). The study of hairy roots mainly involves classification cytology, molecular biology and genetic breeding, and has made some progress (Aghaali et al., 2024; Ajdanian et al., 2024; Karami et al., 2024). However, there is no scientific and systematic collation and summary of researches in these fields with quantitative analyses.

Based on the academic publications in web of science (WOS), this study conducted a comprehensive quantitative assessment of the scientific literature related to hairy root researches, focusing on the period from 2009 to 2024. By using the method of bibliometric analysis, this article discussed the research status and development trend in the past 15 years, aiming to provide basic scientific basis for the optimization of the basic theory and application system of hairy roots. A visual analysis of scientific literature was constructed with CiteSpace and VOSviewer, and the main goals and contributions of this article are as follows: (1) The number of publications and subject information related to hairy root researches were counted and analyzed to predict the future publication trend; (2) The analysis of the co-occurrence relationship between major groups of hairy root researches, understand the geographical distribution characteristics of the field, highlight cooperation and main research directions, to explore potential cooperation opportunities among researchers; (3) Through citation and keyword analysis, the research progress and hot spot trends in this field were determined, and future research topics are proposed.

## 2 Materials and methodology

### 2.1 Data collecting

**Figure 1:**
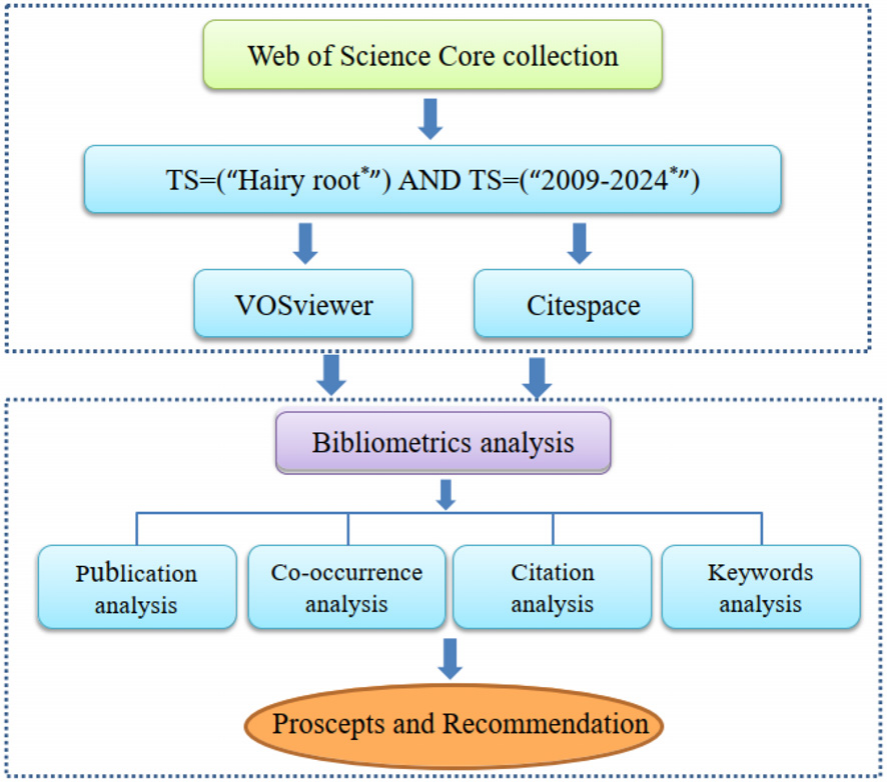
Research working flow chart of this study. ‘‘TS = hairy roots’’ indicated that hairy root was used as the topic for the search and ‘‘TS =2009-2024’’ indicated that the time was span between 2009- 2024.

The retrieval of scientific literature related to hairy roots was extracted based on web of science, which is the largest and most authoritative comprehensive information database (Mongeon and Paul-Hus, 2016). It contains the most influential research literature and is described as one of the most outstanding literature search tools in the world (Vieira and Gomes, 2009). The scientific literature of this topic is limited to January 1, 2009 to December 31, 2024. The starting collecting time is set on January 11, 2024, through “advanced search” of WOS and with “TS = hairy roots” as the searching terms. According to the title and keywords of the article, the search results were further filtered and exported in the format of plain text file. A total of 3935 scientific papers were identified for bibliometric analysis, and the search and analysis flow chart is shown in **Figure 1**.

### 2.2 Research methods

The CiteSpace (version 6.3.R1) and VOSviewer (version 1.6.20) were employed to visualize and analyze the publications from the WOS database. In CiteSpace, “author”, “country”, “institution” and “keyword” are used as key nodes with “January 2009 to December 2024” as time slices. In the analysis, the picture is simplified by the “pathfinder algorithm” with other default parameters. In VOSviewer, the journal coupling analysis was constructed with node types set as “bibliographic coupling, source”. The online websites ChiPlot and BioLadder are used to create histograms and chords.

## 3 Results

### 3.1 Publication information analysis

#### 3.1.1 Publication statistics

**Figure 2:**
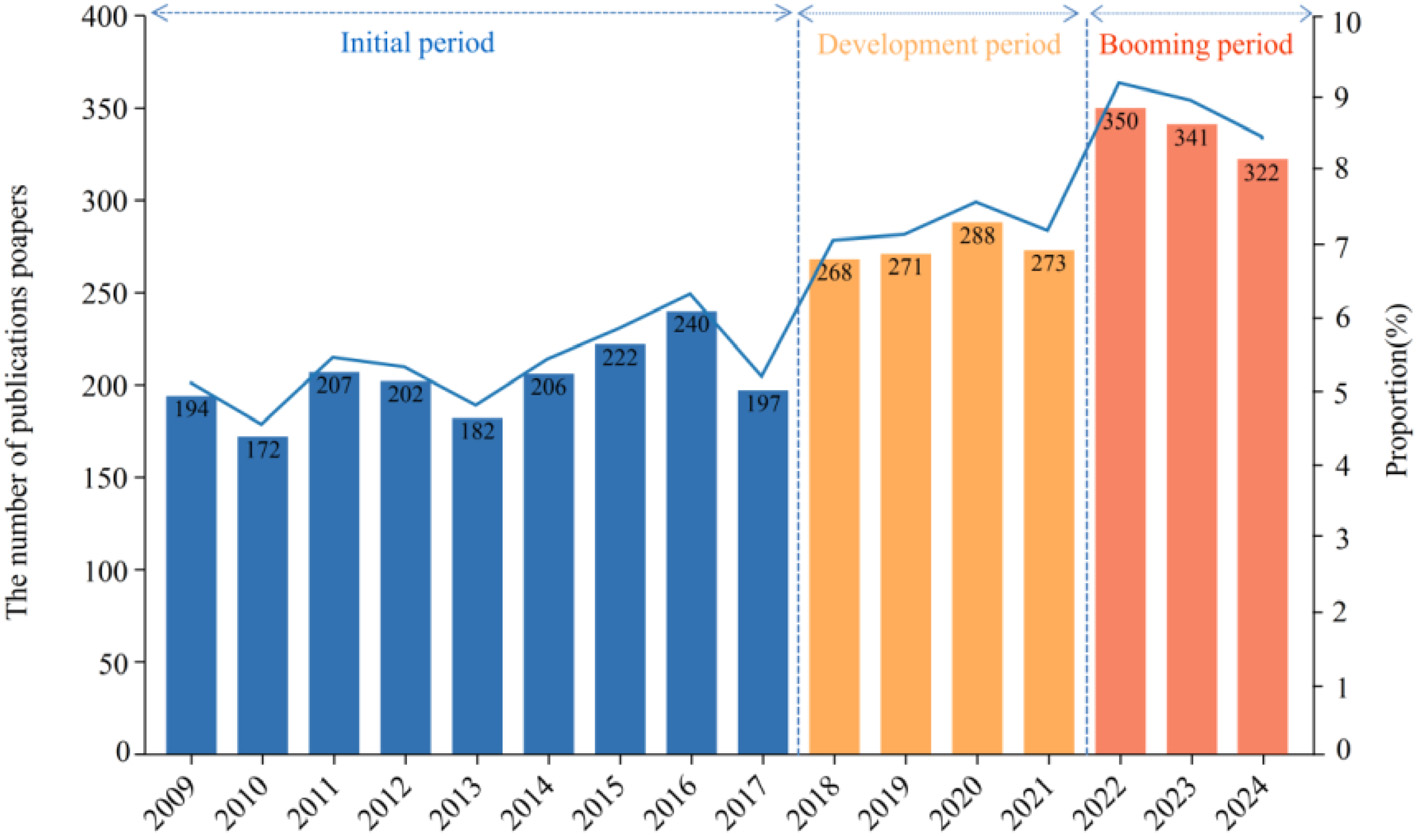
The analysis of annual publication on hairy root researches from 2009 to 2024. The ratio of the annual publication to the total publication is taken as proportion from 2009 to 2024.

Through the retrieval of the WOS database, a total of 3935 non-redundant hairy roots related publications from 2009 to 2024 were used for bibliometric analysis. In general, the description presented in **Figure 2** illustrated an increasing trend of scientific literature. According to the number of publications, the study was divided into three stages, as “Initial period” (2009-2017), “Development period” (2018-2021) and “Booming period” (2022-2024). In the initial stage, the number of articles was 1822, with an average of about 202. The number of publications was 275 per year in the development period. Since 2022, the number of publications has increased significantly, reaching about 337 articles per year. Although the number of publications shows a certain decreasing trend in the booming period, it is in line with the research trend of previous years. Generally, these results highlight the growing academic interest of hairy root research.

#### 3.1.2 Subject categories

**Figure 3:**
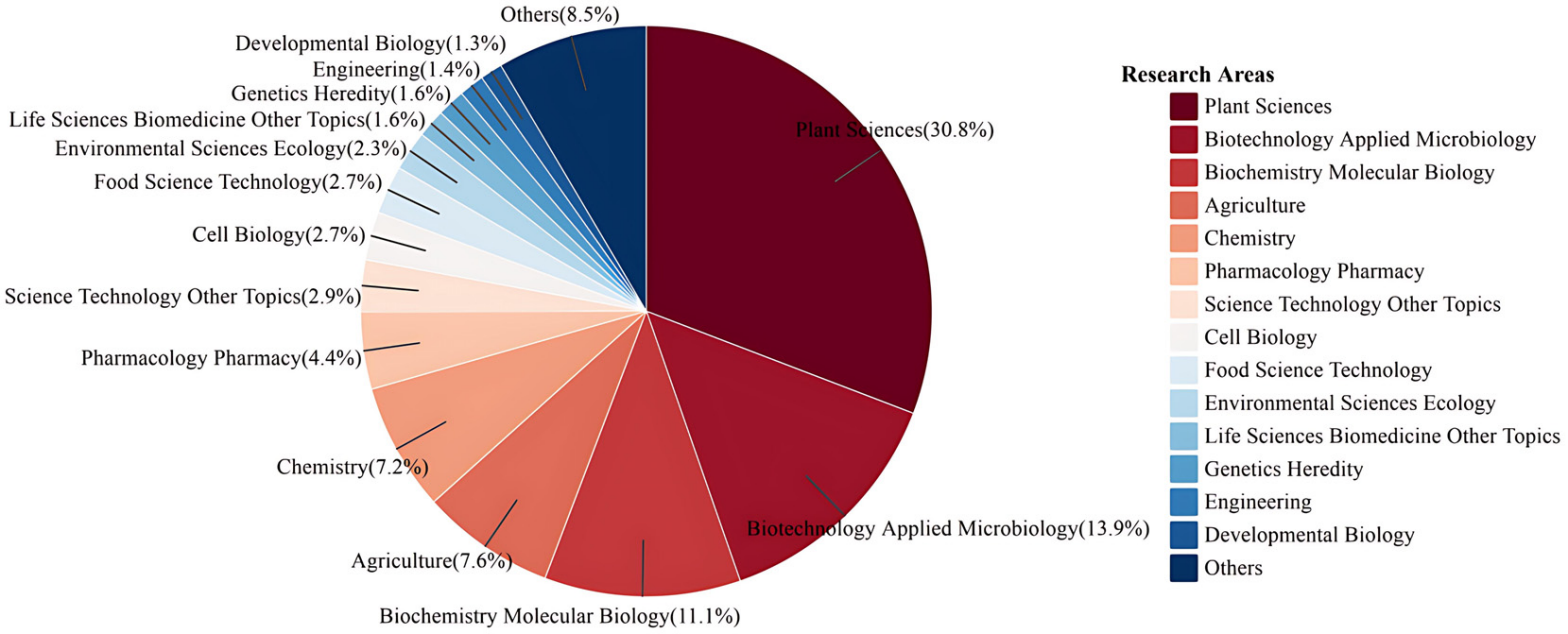
Analysis of the number and distribution of research areas on hairy root.

The statistics and analysis of the disciplinary involved in the publications related to hairy roots were established **Figure 3**. Plant Sciences with 1859 articles accounted for the largest proportion, followed by Biotechnology Applied Microbiology (836 articles) and Biochemistry Molecular biology (598 articles). In addition, Agriculture, Chemistry and Pharmacology Pharmacy also have a relatively high proportion, which is a hot field of hairy root research. Thus, the study of hairy roots revealed the characteristics of multidisciplinary structure and diversity in the fields of plant science, agronomy and chemistry (Banerjee et al., 2012; Ludwig-Müller et al., 2014; Morey and Peebles, 2022). These results suggested that hairy root research has the potential to address global challenges and promote the development of agriculture and bio-industry.

### 3.2 Research profiles

#### 3.2.1 Country contribution

**Figure 4:**
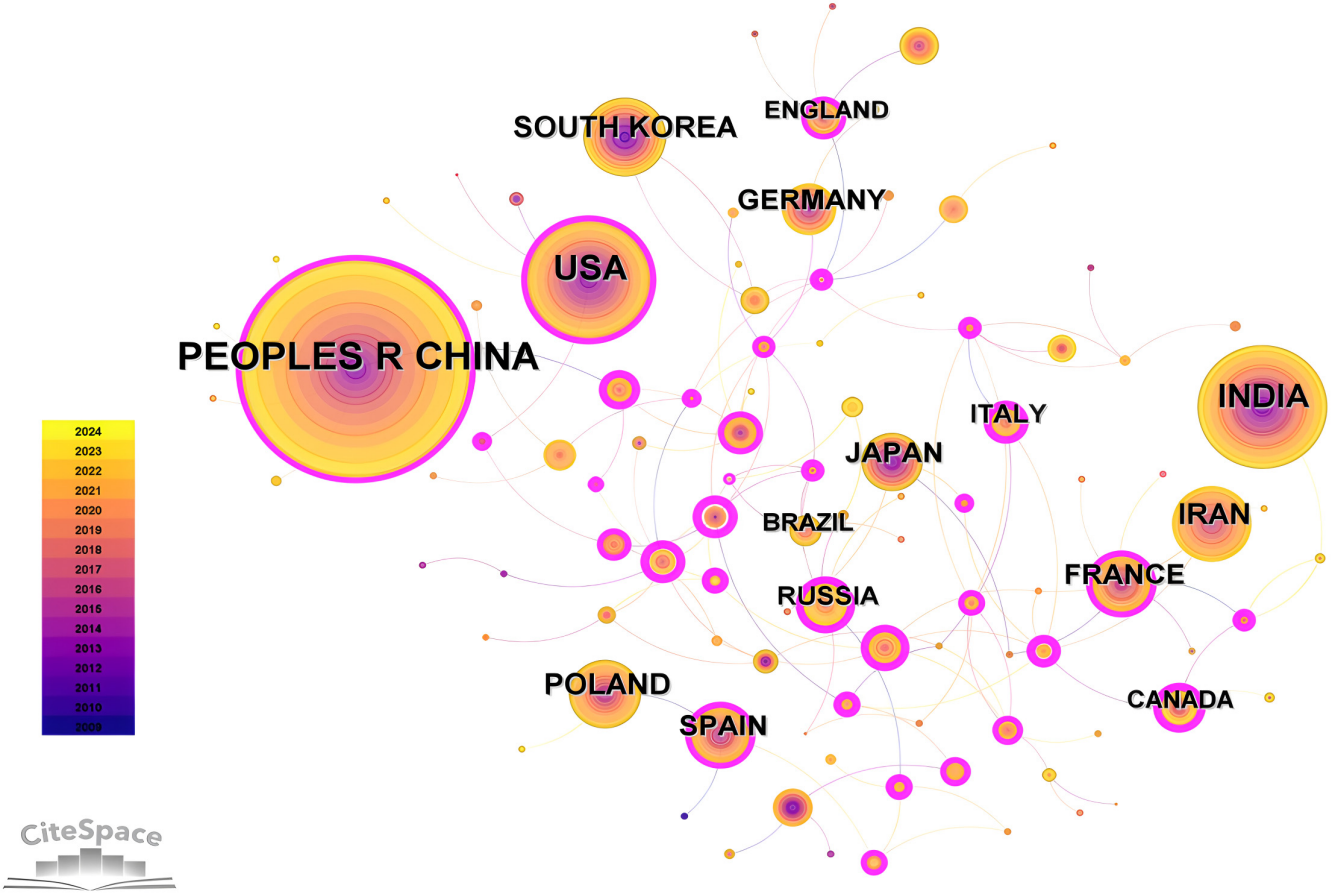
Distribution and frequency analysis of hairy roots in geographical regions. The size of the circle indicates the number of articles and the purple outer circle indicates the centrality.

**Figure 5:**
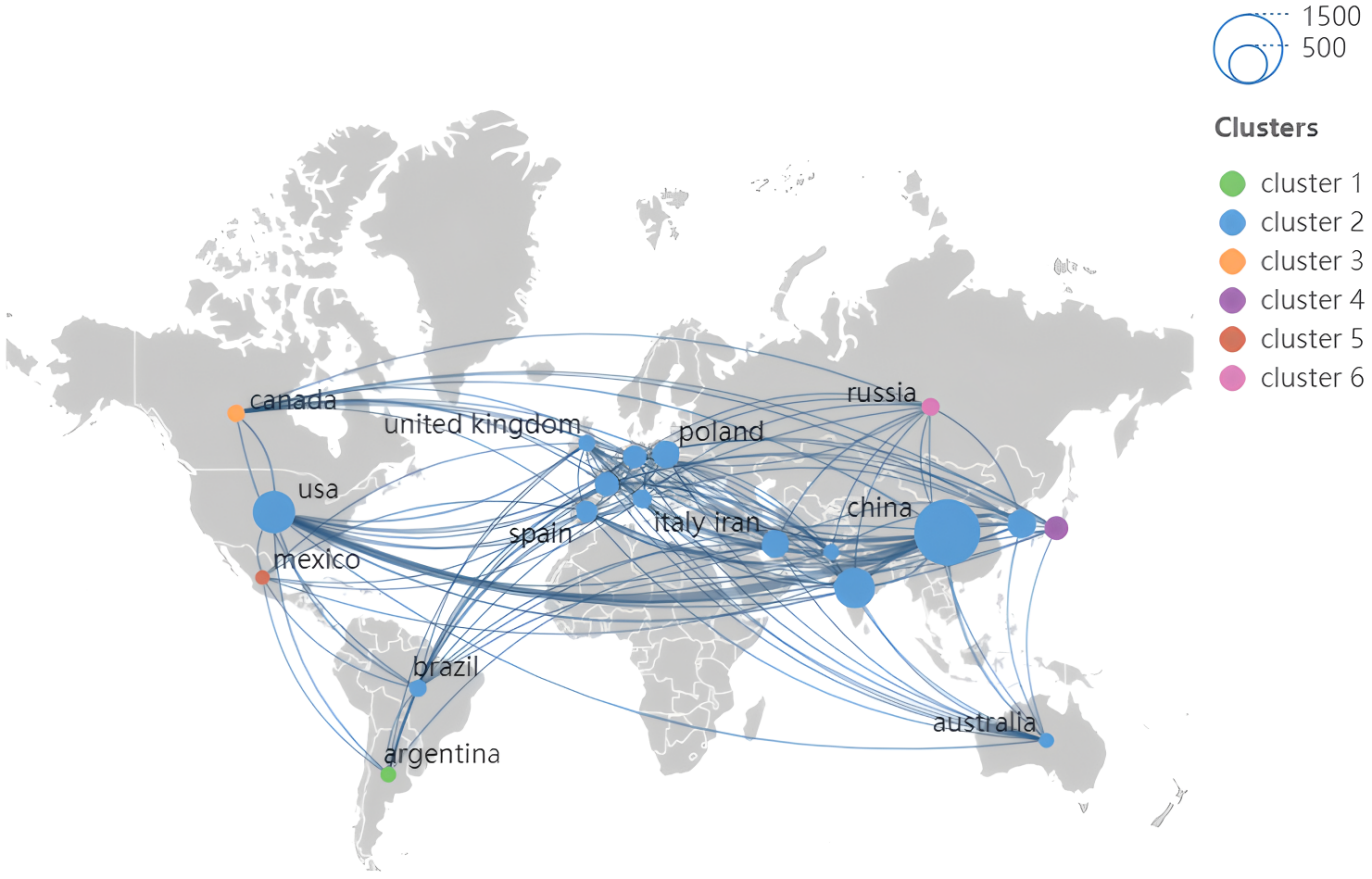
Geographical distribution analysis of national cooperation network based on publication output. The magnitude of the circle represents the number of articles and different colors represent countries in different clusters with the intensity of cooperation.

The number and distribution of publications are analyzed based on the country and shown in the **Figures 4**, **5**; **Table 1**. China has the highest number of publications (1249), accounting for 31.74%, and was the major country, followed by America (511 publications) and India (467 publications). A total of 103 countries participated in the study of this field. Among them, China and USA also have higher centrality values, which illustrated their close cooperation with other countries in this field. In addition, the number of articles in India is high but the centrality is 0.09, indicating less cooperation with other countries. In the national cooperation map of geographic visualization, in Asia, the cooperation between countries is mainly China, South Korea, Japan and India. In North America, such as America and Mexico, cooperation and exchanges are mainly between the countries from European, Asian and South America, including China, Brazil, France and Spain. Among them, China has cooperation with many countries, forming a network of cooperation in Asia, Europe, Oceania and North America. In the field of hairy root research, scientists from different countries should be encouraged to carry out research through interdisciplinary cooperation, sharing resources and technologies.

**Table 1:** Ten countries with the highest number of articles on hairy roots.

Country	Cluster	Links	Centrality	Documents	Citation	Average Citations
China	1	247	0.22	1249	32920	26.36
USA	1	242	0.19	511	18799	36.79
India	2	117	0.09	467	5886	12.60
South Korea	1	96	0.02	236	6235	26.42
IRAN	2	85	0.09	212	4089	19.29
Poland	2	54	0.07	206	3615	17.55
Japan	1	162	0.09	162	4981	30.75
France	2	118	0.26	159	5886	37.02
Germany	1	87	0.13	146	5187	35.53
Spain	3	102	0.1	128	4050	31.64

#### 3.2.2 Contribution of the institutions

**Figure 6:**
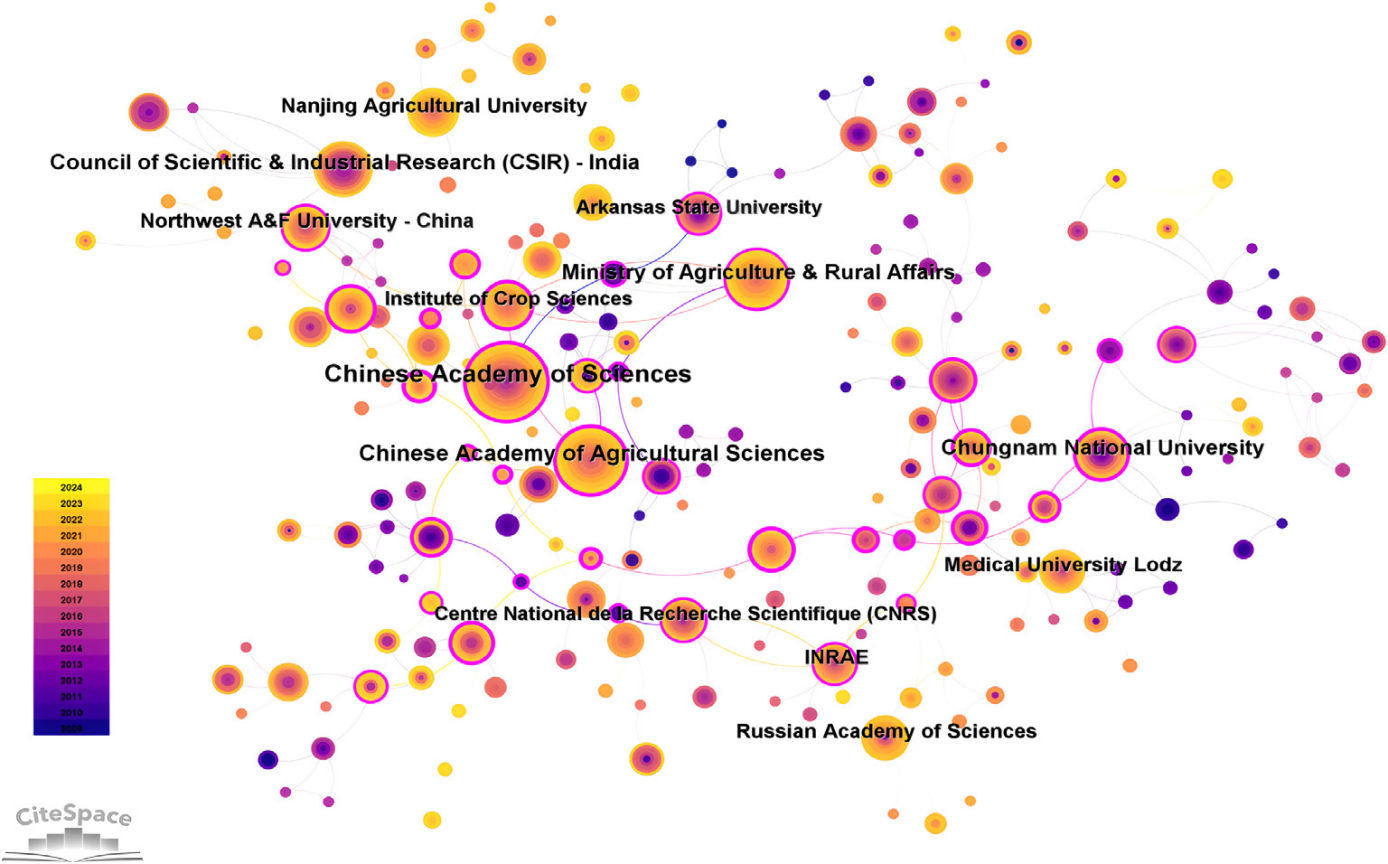
Construction of coexistence network of different institutions from 2009 to 2024.

**Figure 7:**
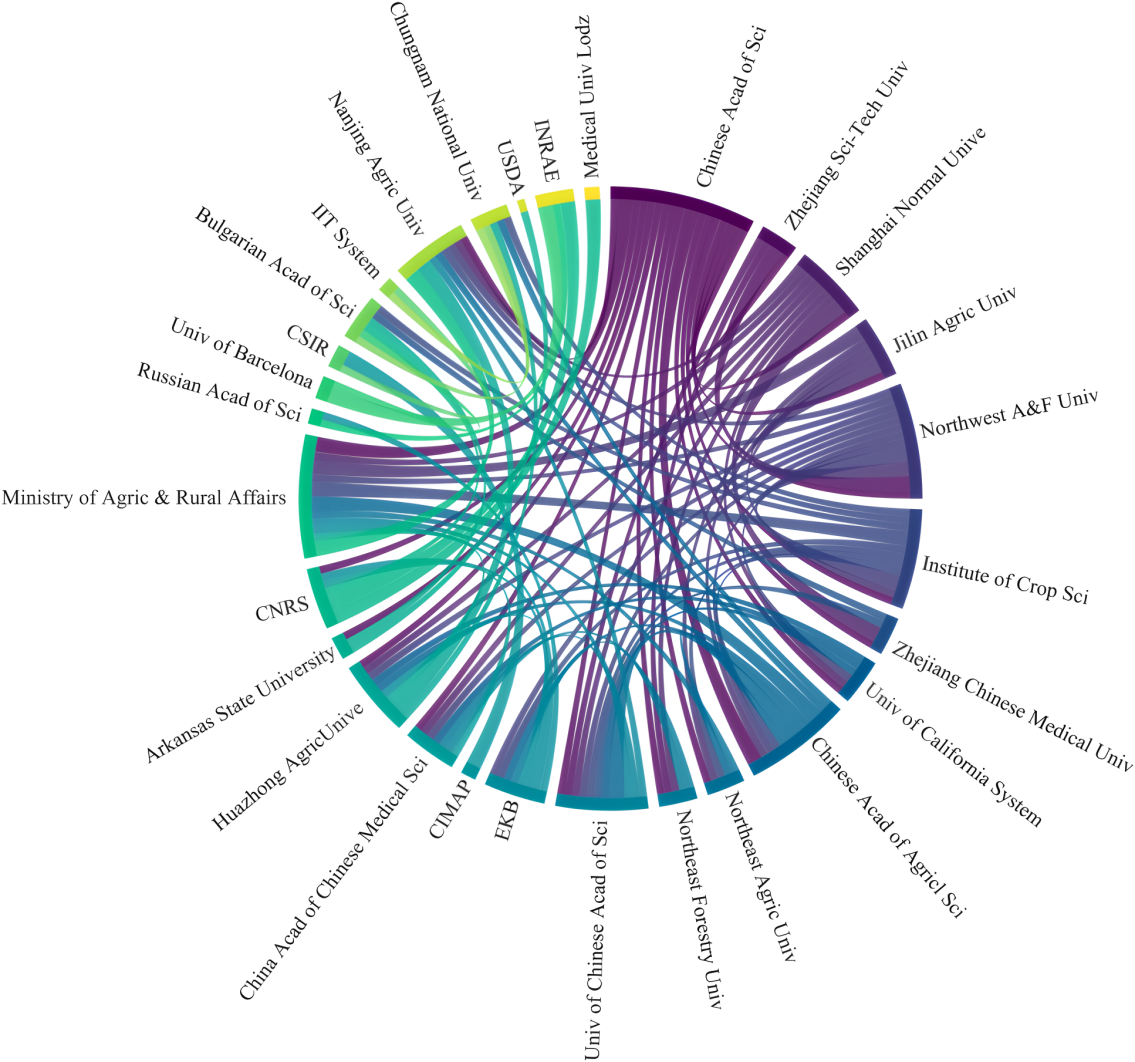
Cooperation between institutions in the field of hairy root research from 2009 to 2024.

Statistical analysis revealed that a total of 288 institutions from 103 countries published articles related to hairy roots **Figure 6**. And among the top ten institutions in the number of articles, five are in China, and the others are in India, South Korea, Russia, Poland and France, respectively. The Chinese Academy of Sciences had the largest number of articles (165, accounting for 4.19%), followed by Chinese Academy of Agricultural Sciences (109 articles), Council of Scientific & Industrial Research (88 articles) and Ministry of Agriculture & Rural Affairs (88 articles). Furthermore, among the top ten institutions, Chinese Academy of Sciences, Chungnam National University and INRAE have high centrality, indicating more collaboration with other institutions. In addition, the cooperation between institutions in the WOS database is generally established in the same country, and there is less external communication. The cooperation relationship among 29 institutions with more than 30 publications was shown in **Figure 7**. For institutions, cooperation in the field of hairy root research is limited, and the Chinese Academy of Sciences has a broader cooperation relationship with other institutions. An enhanced cooperative relation of institutions is essential for the study of hairy roots.

#### 3.2.3 Author collaboration

**Figure 8:**
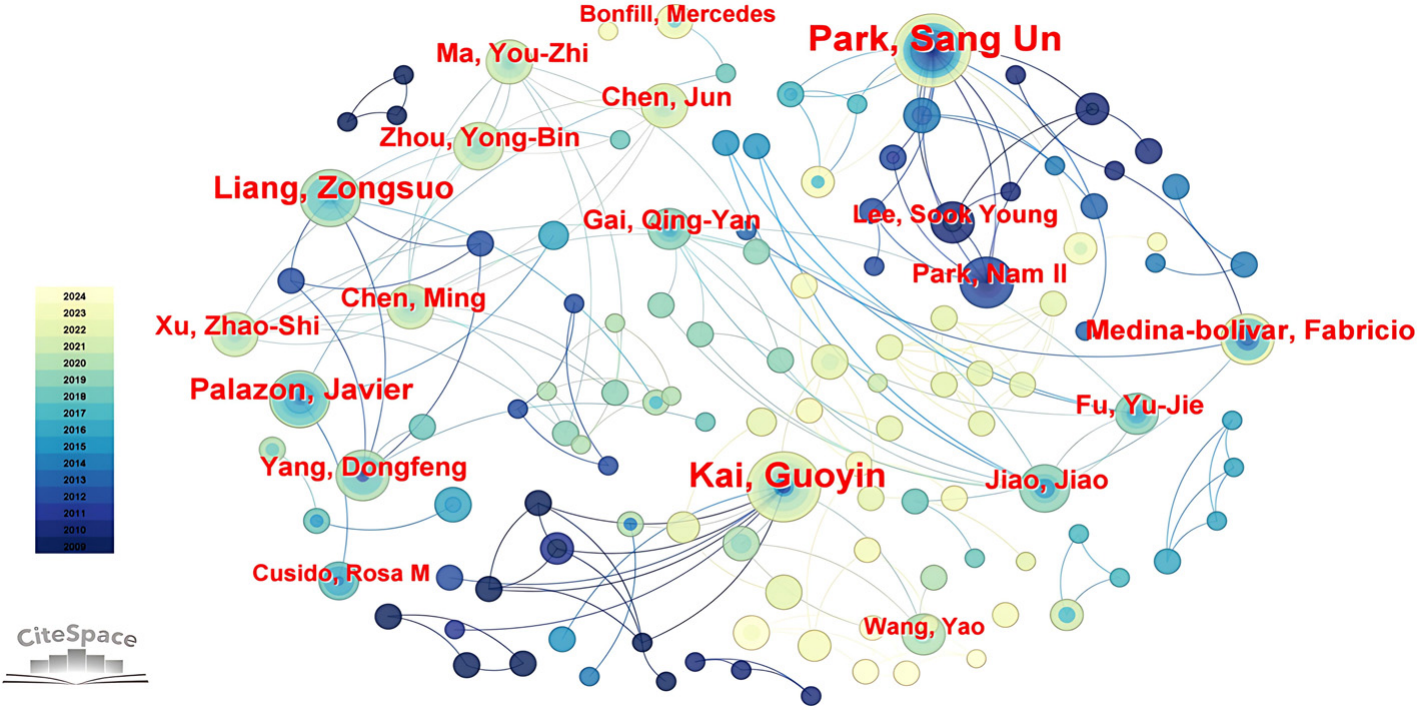
The establishment of author co-occurrence network in WOS database.

The top 10 authors in terms of the number of publications are shown in **Table 2** and the top researchers are mainly Park SangUn (67 articles, Chungnam National University), Kai Guoyin (54 articles, Zhejiang Chinese Medical University) and Liang Zongsuo (48 articles, Zhejiang Sci-Tech University). The cooperative relationship between the authors was further analyzed in **Figure 8**, Kai Guoyin, Xu ZhaoShi and Chen Ming have collaborated more closely with other authors. In general, there is relatively little cooperation and communication between different authors.

**Table 2:** The top 10 most productive authors in hairy root research from 2009 to 2024.

Code	Author	Documents	Citations	institution	Country
1	Park SangUn	67	1046	Chungnam National University	South Korea
2	Kai Guoyin	54	2817	Zhejiang Chinese Medical University	China
3	Liang Zongsuo	48	1825	Zhejiang Sci-Tech University	China
4	Palazon Javier	48	1663	University of Barcelona	Spain
5	Medina-bolivar Fabricio	40	433	Arkansas State University	America
6	Agostini Elizabeth	32	743	National University of Río Cuarto	Argentina
7	Yang Dongfeng	31	1037	Zhejiang Sci-Tech University	China
8	Huang luqi	30	1718	China Academy of Chinese Medical Sciences	China
9	Zhang Lei	25	1084	Naval Medical University	China
10	Jiao Jiao	23	524	Northeast Forestry University	China

**Figure 9:**
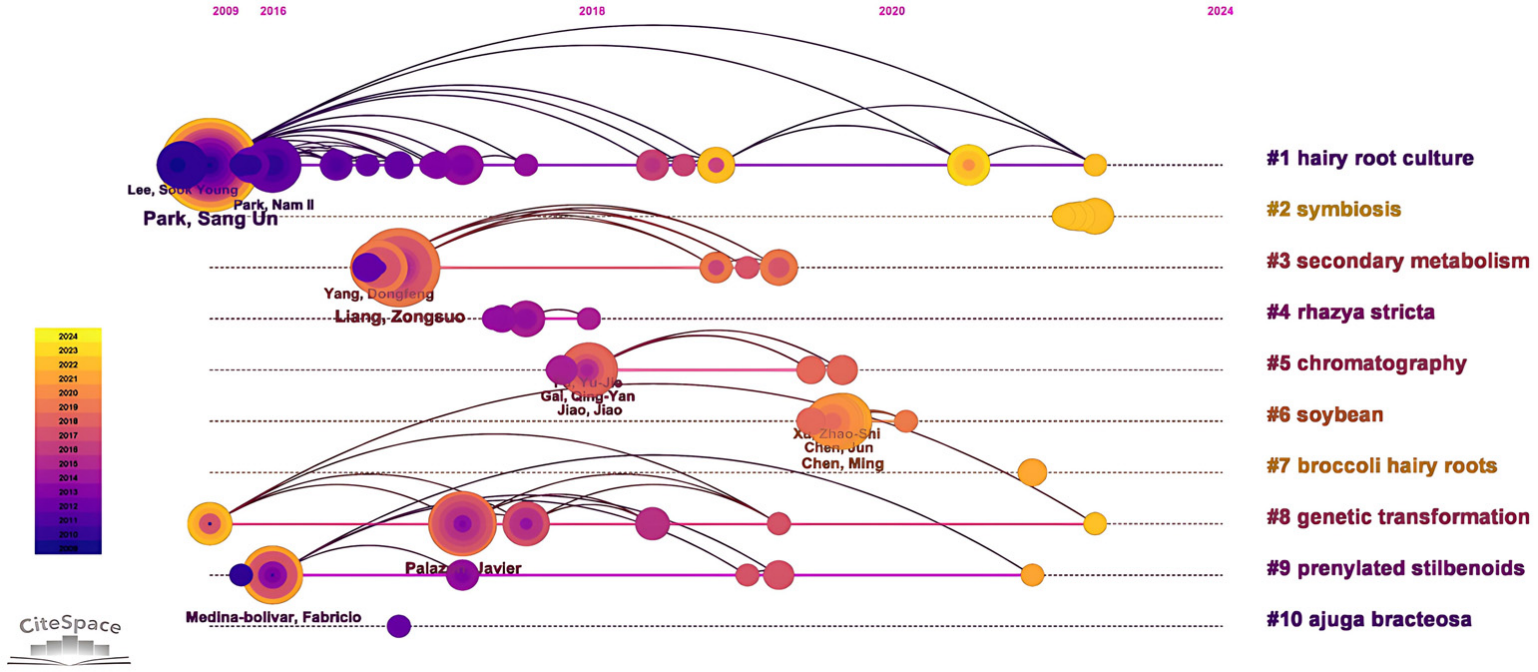
Timeline diagram of author collaborative based on keyword clustering.

According to keyword clustering, a timeline diagram of author collaborative was constructed **Figure 9**, and the study of researchers on hairy roots mainly focused on the fields related to hairy root culture, secondary metabolism and genetic transformation (El-Esawi et al., 2017; Xue et al., 2024). Among them, Xu Zhaoshi paid more attention to the study of hairy roots in soybean field. On the whole, there are some differences in the topics of hairy root research among the authors of different institutions. These results indicated that hairy root research has a multidisciplinary structure and diversity, and researchers should integrate resources and technologies in different fields to promote the research and application of hairy roots.

#### 3.2.4 Journal analysis

**Figure 10:**
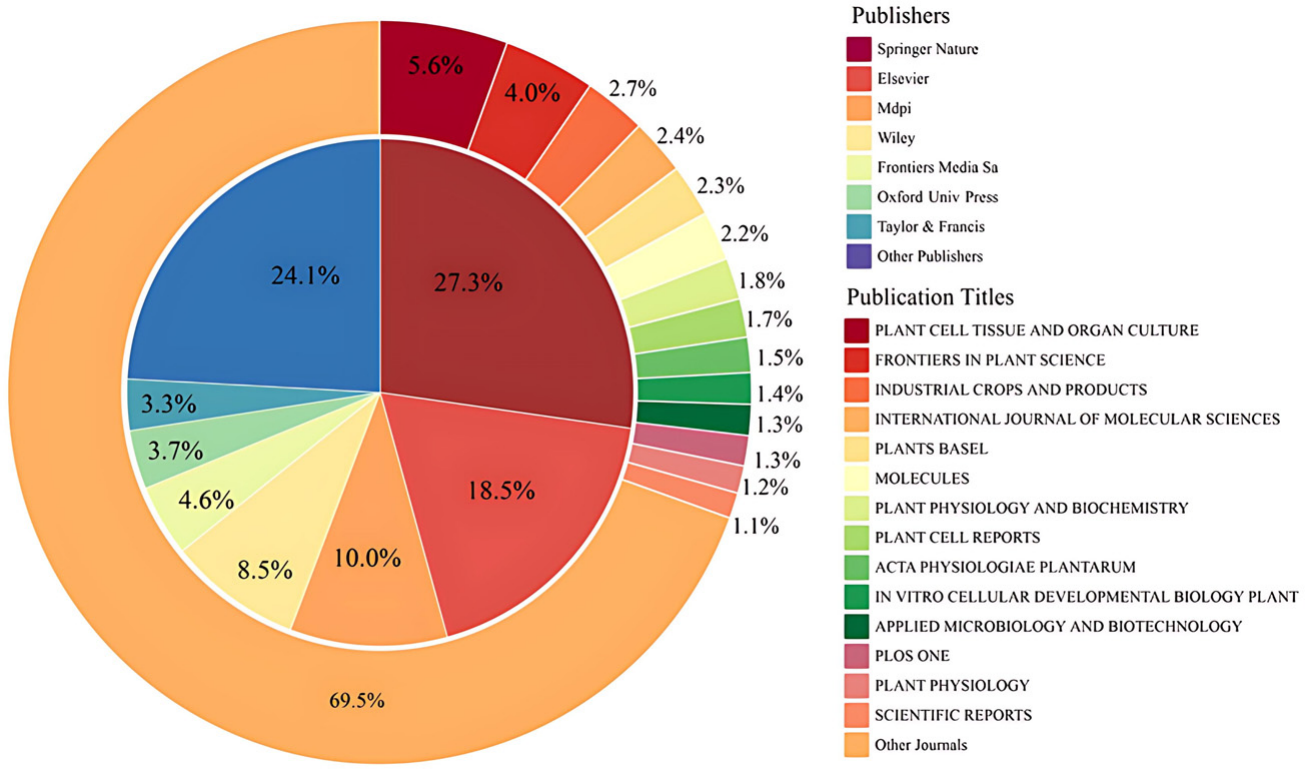
The major publishers and publication titles for hairy roots from 2009 to 2024. The doughnut diagram represents the type and proportion of the publication title, and the internal pie chart represents the type and proportion of the publisher.

**Figure 11:**
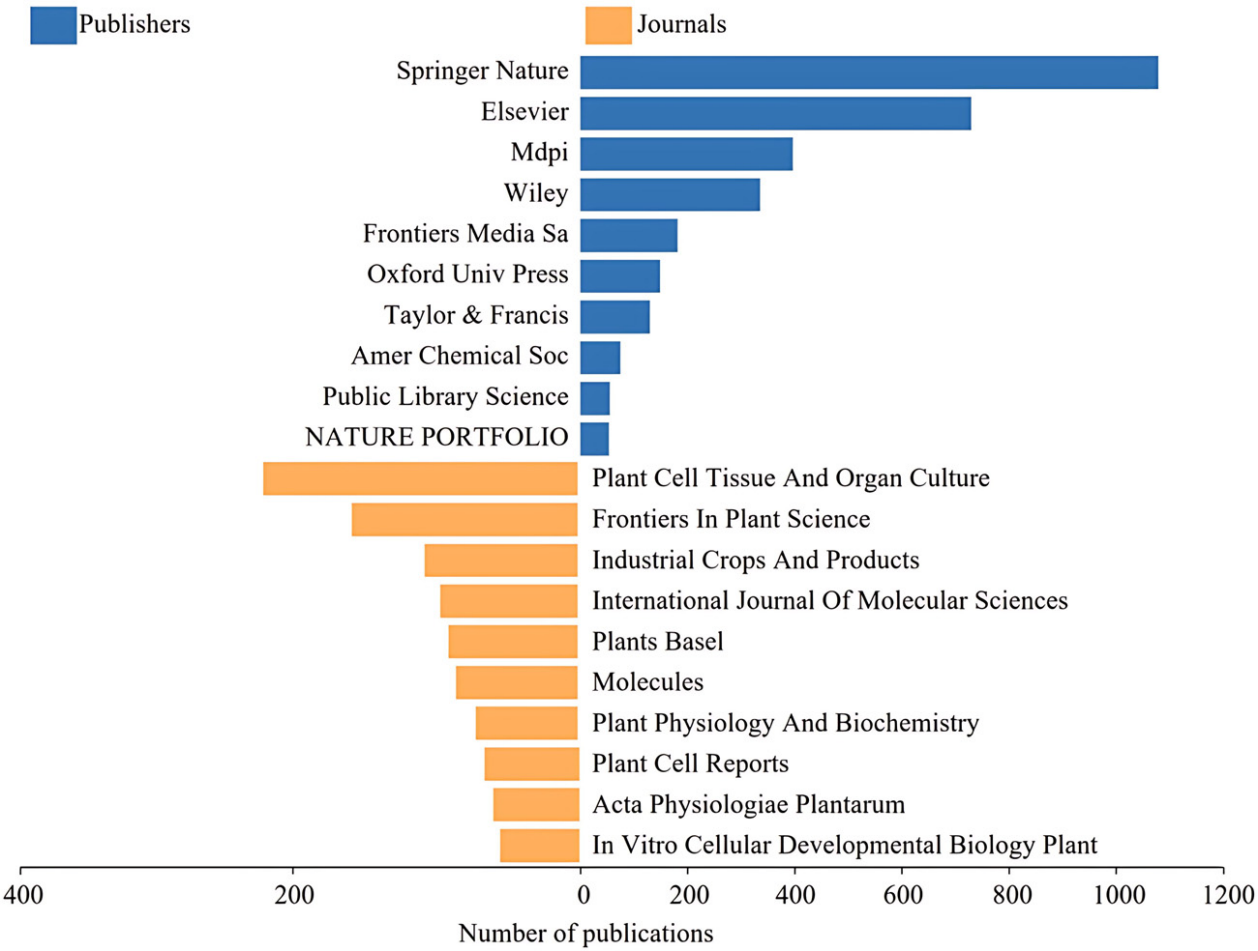
The top 10 journal sources and publishers.

**Figure 12:**
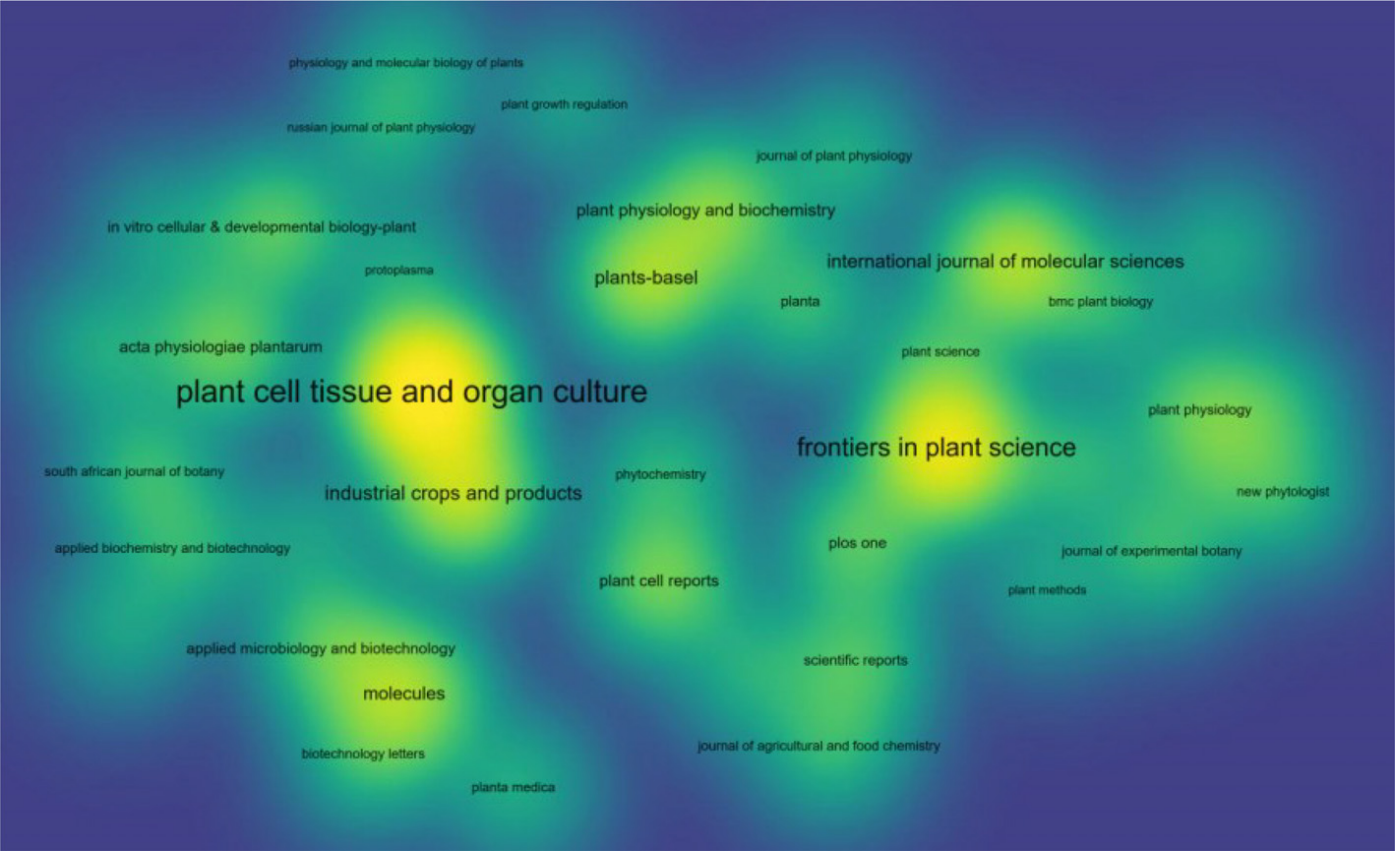
Density plot of coupling analysis of journal citations in hairy root research. The node size represents the number of publications in the journal.

Further analysis of the sources of publications from the WOS database **Figures 10**–**12**, the publishers with the largest number of articles on hairy root research are mainly Springer Nature, Elsevier, Mdpi, Wiley and Frontiers Media Sa. Besides, according to the number of publications, the top-ranked was Plant Cell Tissue and Organ Culture, accounting for 4.19%, followed by Frontiers in Plant Science, Ndustrial Crops and Products. The above journals mainly involve the field of plant science, focusing on plant physiology.

### 3.3 Research hotspot and trend

#### 3.3.1 The most cited publications

**Figure 13:**
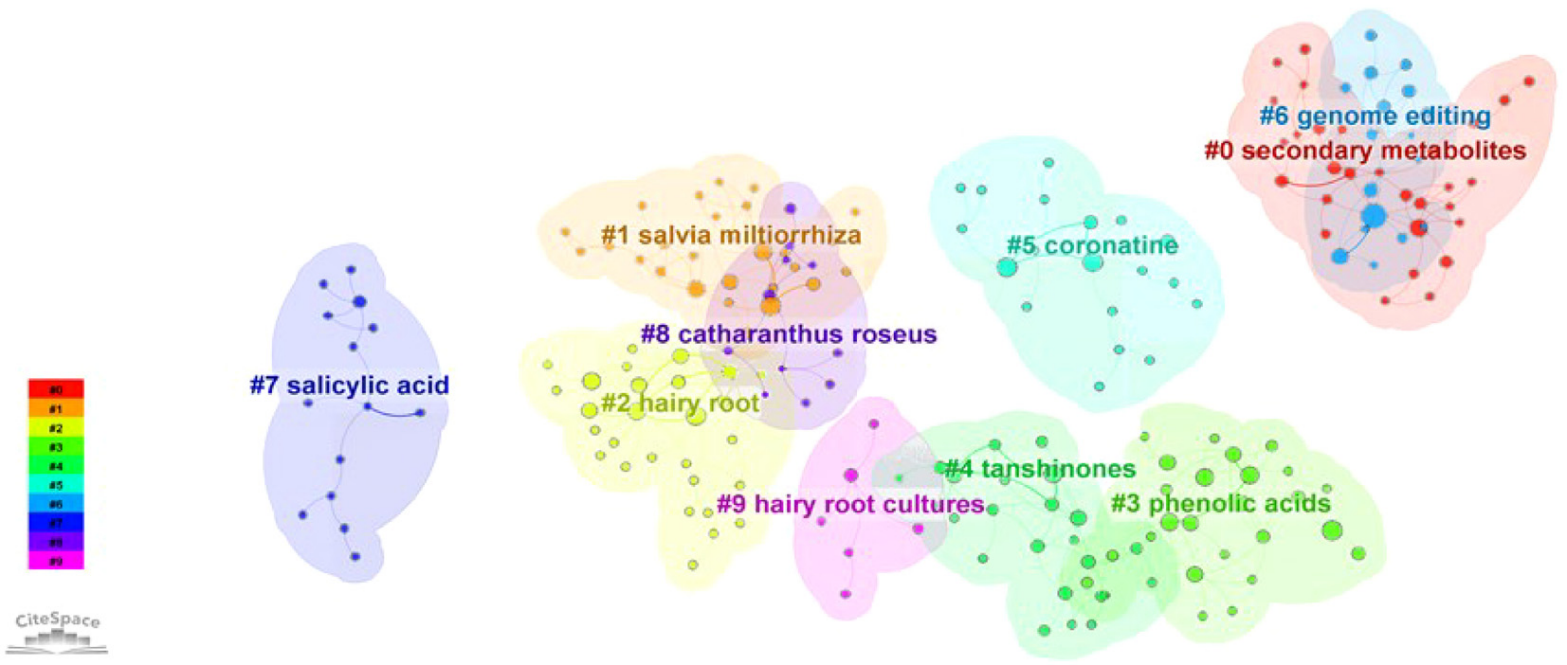
Publications clustering analysis based on keyword.

Ranking these publications by citation frequency and finding that the most cited articles are mainly related to research such as plant science. The information of the top ten most cited publications is listed in **Table 3**. The most cited publications were mainly related to genetic transformation and secondary metabolic synthesis. Further, a cluster analysis of these articles was established based on keywords, and they were divided into 9 clusters, including assalicylic acid, salvia miltiorrhiza, coronatine, secondary metabolism, genome editing and hairy root culture (**Figure 13**). The above results indicate that the study of researchers on hairy roots focuses on genetic transformation, stress response and secondary metabolism.

**Table 3:** The top ten most cited publications on hairy root research from 2009 to 2024.

Code	Title	Author	Year	TLCS
1	Metabolic engineering tanshinone biosynthetic pathway in Salvia miltiorrhiza hairy root cultures	Guoyin Kai	2011	230
2	Bioactivities, biosynthesis and biotechnological production of phenolic acids in Salvia miltiorrhiza	Min Shi	2018	205
3	The Transcription Factor CrWRKY1 Positively Regulates the Terpenoid Indole Alkaloid Biosynthesis in Catharanthus roseus	Nitima Suttipanta	2011	299
4	Antioxidant Activity and Phenolic Content of Betalain Extracts from Intact Plants and Hairy Root Cultures of the Red Beetroot Beta vulgaris cv. Detroit Dark Red	Vasil Georgiev	2010	240
5	Verbascoside — A review of its occurrence, (bio)synthesis and pharmacological significance	Kalina Alipieva	2014	330
6	Targeted mutagenesis in soybean using the CRISPR-Cas9 system	Xianjun Sun	2015	261
7	Hairy Root Transformation Using Agrobacterium rhizogenes as a Tool for Exploring Cell Type-Specific Gene Expression and Function Using Tomato as a Model	Mily Ron	2014	240
8	*In vivo* grapevine anthocyanin transport involves vesicle-mediated trafficking and the contribution of anthoMATE transporters and GST	Camila Gomez	2011	212
9	Nod Factor/Nitrate-Induced CLE Genes that Drive HAR1-Mediated Systemic Regulation of Nodulation	Satoru Okamoto	2009	297
10	Plant cell culture strategies for the production of natural products	Ochoa-Villarreal	2015	196

#### 3.3.2 Keyword analysis

**Figure 14:**
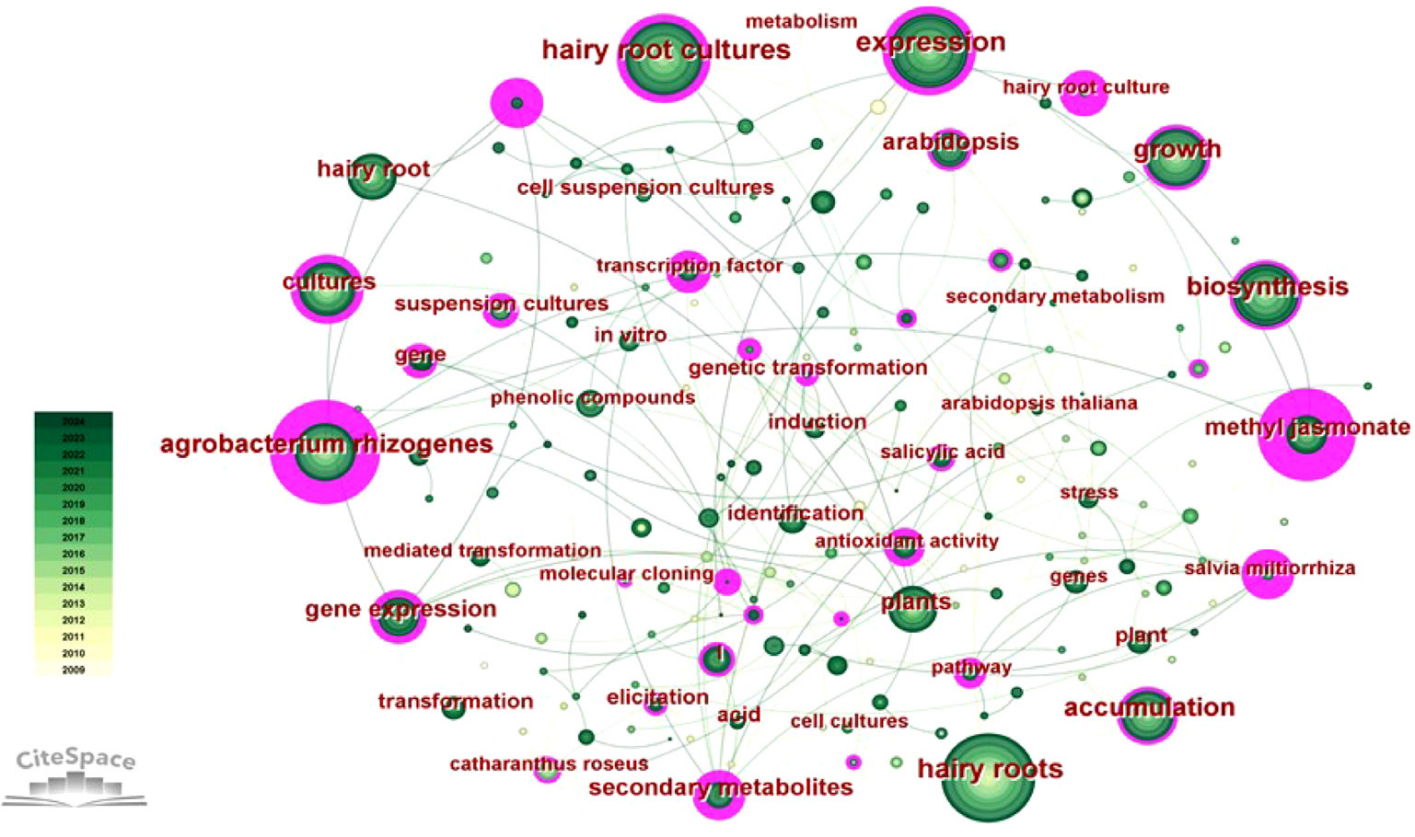
The co-appearance network of keywords.

As a condensed summary of the article, keywords are the main direction of the research content and can be used as a key indicator to identify related research. In CiteSpace, a collinear network of keywords for all publications related to hairy root research was constructed as **Figure 14**. A total of 5 keywords with a frequency greater than 500 were retrieved, including hairy roots, hairy root cultures, expression, growth, agrobacterium rhizogenes. In addition, the five keywords with the highest centrality were secondary metabolites, gene expression, accumulation, hairy root cultures, agrobacterium rhizogenes. These keywords with high frequency and centrality reveal the theme and development direction of hairy root research.

#### 3.3.3 Analysis of burst words

**Figure 15:**
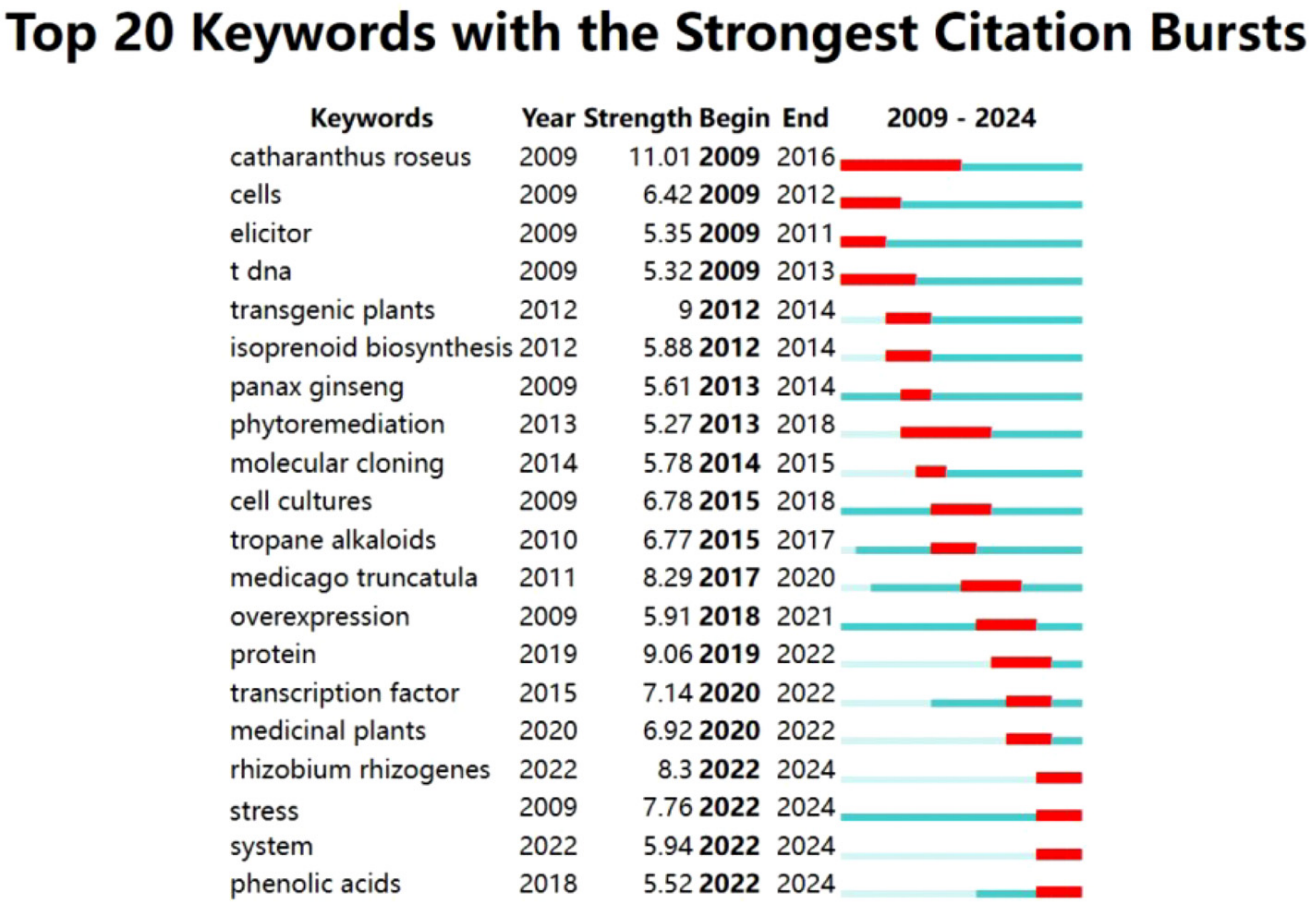
Top 20 keywords in terms of citation bursts of hairy root research.

As shown in the **Figure 15**, in CiteSpace, keywords were further analyzed through the burst analysis function to predict potential research fronts. During the period from 2009 to 2014, the keywords mainly included T-DNA, cells and elicitor, which indicated that the research on hairy roots during this period focused on the induction of hairy roots. In the next stage (2012-2018), the key words of hairy root research mainly include transgenic plants, cell cultures and tropane alkaloids, indicating that hairy root research has transitioned to scientific application. Since 2018, high-intensity burst words include overexpression, protein, transcription factor and genes. This change indicates that researchers have focused on the relationship between hairy roots and the molecular mechanism of plant functional genes.

**Figure 16:**
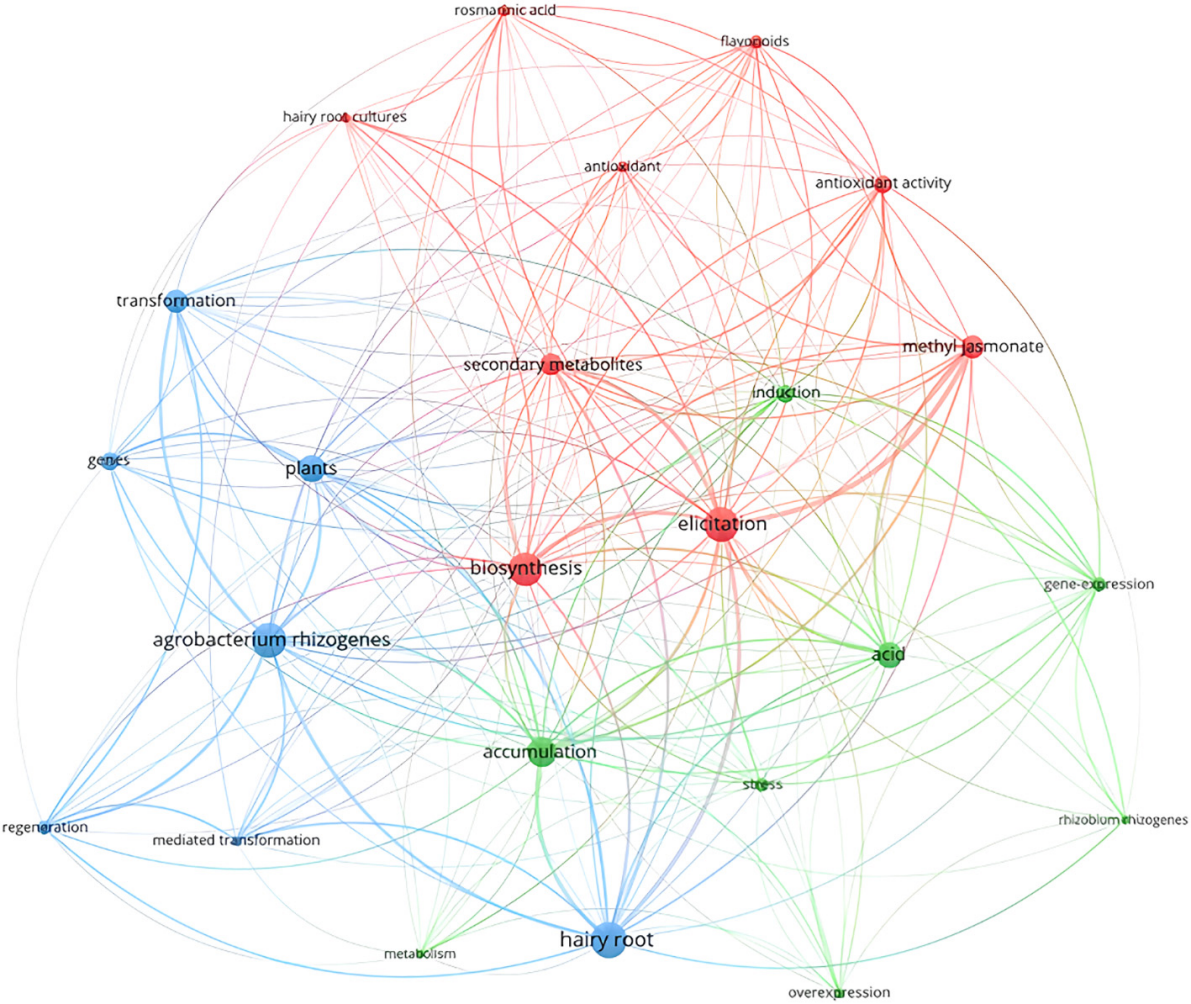
The co-appearance network of keywords form 2022-2024.

To further explore the potential trends in the field of hairy root research, the keywords of the literature from 2022 to 2024 were analyzed **Figure 16**. These keywords are mainly related to genetic transformation and substance synthesis, including secondary metabolism, genes, elicitation, transform, flavonoids and biosynthesis. The keywords with collinearity indicate that the research in the field of hairy roots has been closely related to genetic transformation and secondary metabolism in recent years. Among the publications related to hairy root research in the past three years, 178 and 121 references 1 were related to genetic transformation and compound synthesis, respectively. Combining the analysis of burst words, the verification of plant functional genes through hairy root-mediated genetic transformation system and the synthesis of secondary metabolites are the current and future research hotspots.

Furthermore, based on the WOS database, the contribution of hairy roots in genetic transformation and secondary metabolism research was analyzed. We only focused on the top fifteen species in the most cited publications. As shown in **Table 4**, as a traditional medicine, the genes and pathways related to secondary metabolism in hairy roots of Salvia miltiorrhiza are described in detail, and the genetic transformation mediated by hairy roots provides a strategy to solve the content limitation of main compounds in Salvia miltiorrhiza. In addition, researchers have also focused on the application of hairy roots in other plants, including *Catharanthus roseus*, *Echinacea purpurea*, and *L. album*. Therefore, the hairy root system can be established by *Rhizobium rhizogenes* to realize the industrial production of secondary metabolites and solve the problem of market demand in the future.

**Table 4:** Analysis of genetic transformation and secondary metabolic publications based on hairy roots.

Species	Genes	Secondary metabolites	Reference
*Salvia miltiorrhiza*	SmERF1b, SmCPS1, SmKSL1, SmERF1b-like, Sm4CL2, SmJAZ8, SmMYB98, SmbHLH10,	tanshinone I, cryptotanshinone, salvianolic acids	Zhang et al., 2015; Pei et al., 2018; Tan et al., 2023; Li et al., 2024; Hao et al., 2018; Xing et al., 2018
*Catharanthus roseus*	1-deoxy-D-xylulose synthase and geraniol-10-hydroxylase or anthranilate synthase, Orca, Zct, ORCA3	terpenoid indole alkaloid	Peebles et al., 2011; Goklany et al., 2013; Sun and Peebles, 2016
*Aristolochia manshuriensis*	rolB, rolC	magnoflorine, aristolochic acids I, aristolochic acids II	Shkryl et al., 2023
*L. album*	PAL,TAL	Flavonoids, lignans	Kumar et al., 2012; Soltani et al., 2023
*Cajanus cajan* (L.) Millsp.	CHS, STS	Flavonoids, cajaninstilbene acid	Jiao et al., 2020; Gai et al., 2022
*Papaver orientale* L.	COR, SalAT, SalR, T6ODM, CODM, Salsyn	Morphine, thebaine	Hashemi and Naghavi, 2016
*Lithospermum erythrorhizon*	LeACS - 1	shikonin	Chandra and Chandra, 2011; Fang et al., 2016; Tatsumi et al., 2016
*Ocimum basilicum* L.	AUX2	rosmarinic acid, antioxidants	Srivastava et al., 2016
*Brassica rapa ssp*.	BrMYB28, BrMYB29, BrMYB34, BrMYB51, BrMYB122, CYP79, CYP83	flavonols, hydroxybenzoic, hydroxycinnamic acids	Chung et al., 2016
*Echinacea purpurea*	PAP26, APase	chicoric acid, alkamide	Abbasi et al., 2009; Romero et al., 2009; Salmanzadeh et al., 2020
*Mentha* sp*icata* L.	PAL, C4H, 4CL, HPPR	phenolic acids	Yousefian et al., 2020

## 4 Discussion

### 4.1 Overview of hairy root research based on WOS database

The results described the research progress of hairy root-related publications, including the progress in the number and impact of publications, as well as the collaboration network between specific countries, institutions and authors. By analyzing the co-occurrence of keywords, the changes in the pattern of hairy root research and its potential to provide information for future research were revealed. As the data revealed, the research field of hairy roots was expanding rapidly, which explained the attention of researchers to hairy roots and its potential for future science and production.

A major result of bibliometric analysis was the high level of collaboration in hairy root research. The research center was mainly located in the countries of China and Western Europe. Among them, China, America, Spain, Australia and Brazil have established intensive cooperation networks and expanded their research fields from Asia to Europe, North America, South America and Oceania. This interconnection not only contributes to the sharing of research resources, but also promotes interdisciplinary innovation, and accelerates the dissemination of knowledge through cooperation in different regions (Salmerón-Manzano et al., 2020; Taghouti et al., 2022). In this field, cooperation between different institutions was mainly established in the same country or region. The Chinese Academy of Sciences was a major contributor to the study of hairy roots. It not only had a high number of publications, but also has close cooperation with institutions in different countries. The close collaboration between the countries was supported by the growth of shared technologies, and this global partnership enables researchers to gain access to hairy root applications in a wider range of plant species.

In addition, the trend in the field of hairy root research was increasingly turning to genetic transformation techniques, secondary metabolic synthesis and verification of plant functional genes. *Rhizobium rhizogenes*-mediated hairy root transformation methods have continuously completed the establishment of genetic transformation systems in various plants, which had greatly promoted the development of plant science (Brijwal and Tamta, 2015; Qin et al., 2021).

Although this study provided valuable insights into the global trend of hairy root research, there were inherent limitations in bibliometric analysis. One of the main limitations was that the study was only based on the WOS database, and the scope of the database used for analysis was very limited, excluding other related studies available in databases with unique advantages, including PubMed, Google Scholar, and CNKI Database. In particular, non-English publications may be neglected, which potentially limits the global diversity of literature, which may lead to research results biased towards regions dominated by English publications (Pranckutė, 2021; Bakhmat et al., 2022).

### 4.2 Research prospects of hairy roots

Since 2009, the focus of research in the field of hairy roots has shifted from hairy root culture to secondary metabolism, plant genetic transformation, medicinal plants and molecular functions. The application of hairy roots focuses on plant genetic transformation, environmental remediation and secondary metabolite accumulation, and plant genetic transformation serves the study of medicinal plants. Broadly speaking, medicinal plants include plant resources used as nutritional agents, certain hobbies, condiments, pigment additives, and pesticides and veterinary drugs with special secondary metabolites.

For medical workers, the results of this study highlighted the potential for medicinal plants to become more widely integrated into healthcare. In recent years, increasing attention to bioactive compounds has shown that plant extracts can play a key role in complementary and integrated medicine as a natural substitute for synthetic drugs (Vichakshana et al., 2022; Abdallah et al., 2023; Hao et al., 2020). To date, the development of medicinal plants is faced with the shortage of plant resources, and the research of hairy root technology can maximize the yield of bioactive compounds and ensure the sustainability of development (Jachak and Saklani, 2007; Li and Wang, 2021; Chaachouay and Zidane, 2024). In addition, although the number of publications in China, America and India dominates, the potential of biodiversity-rich regions, such as parts of Africa and South America, in the study of medicinal plants and hairy roots is not fully utilized. From the perspective of decision-making, by providing funds and encouraging international partnerships, it can contribute to the local economy and globalize research objectives (Azwanida, 2015).

In recent years, with the rapid development of gene editing technologies such as CRISPR-Cas9, the application potential of hairy root system in metabolic engineering, functional genomics and medicine development has been further released, showing unique advantages and broad prospects (Cai et al., 2015; Niazian et al., 2022). Hairy roots naturally have the ability to synthesize a variety of high-value secondary metabolites (such as alkaloids, terpenoids, and phenolic compounds), but their yield is often limited by the expression level of endogenous genes (Yousefian et al., 2020; Li et al., 2024). Gene editing technology can significantly increase the accumulation of target products by targeted knockout of inhibitors in metabolic pathways or activation of key rate-limiting enzyme genes (Jedličková et al., 2024). The combination of genetic plasticity of hairy roots and gene editing technology provides an ideal chassis for synthetic biology, and can also accelerate functional genomics research and break through the limitations of traditional breeding (Cheng et al., 2021). Despite the great potential, the application of gene editing in hairy roots still faces technical bottlenecks, such as species dependence of editing efficiency, complexity of multi-gene synergistic regulation, and maintenance of metabolite stability in large-scale culture. The above problems need to be solved urgently to further improve the accuracy and industrialization feasibility of the hairy root system.

Furthermore, the problems of global environmental pollution and ecological balance destruction have become increasingly prominent, and hairy roots have shown considerable potential in environmental stress (Khin et al., 2012; Agostini et al., 2013). Compared with traditional remediation methods, bioremediation has the advantages of safety, low cost, reusability and easy operation. Hairy roots have significant effects in the field of environmental remediation, especially in the enrichment of heavy metal elements, adsorption of phenolic compounds, and removal of nitrogen-containing dyes (Zhou et al., 2013). For example, the accumulation of cadmium in the hairy roots of rape increased with the increase of cadmium concentration, indicating the characteristics of enrichment of heavy metal cadmium, which can be used as an excellent cadmium tolerance test material for cadmium enrichment. In tomato, hairy roots can effectively adsorb and remove phenols from water. In summary, the application of hairy roots in the field of environmental remediation has made some progress, and future research in this area will receive more and more attention (González et al., 2008; Dixon and Dickinson, 2024).

Although the industrialization of hairy roots is still subject to some limitations, the optimization conditions of hairy roots of different species vary greatly; susceptible to bacterial or fungal contamination during long-term culture; efficient extraction of metabolites from complex root tissues requires additional steps and costs; the market demand for high-value metabolites (such as anti-cancer drugs) is limited, while the production of bulk chemicals tends to choose lower-cost systems. Hairy roots have advantages in saving land resources, growth rate and the ability to synthesize secondary metabolites. Combined with the interdisciplinary technology integration, it will continue to overcome the limitations in the process of industrialization.

## 5 Conclusion

The increasing importance of hairy root research has been described, especially their potential in drug synthesis, therapeutic applications and environmental remediation. Based on the WOS database, this study makes a bibliometric analysis of 3935 papers published from 2009 to 2024, and discussed the research development, hot spots and frontier trends in this field. The main conclusions were as follows: In recent decades, the study of hairy roots had shown a slow growth trend, with 3935 articles published from 2009 to 2024. Asia and Western Europe were the centers of hairy root research. Among them, China, America and India dominate the publication output, and China had established cooperative relations with many countries in South America, Europe, Oceania and Asia. The research institutions were mainly universities, and the Chinese Academy of Sciences was the major research institution, but the cooperation between different institutions needs to be strengthened. Park Sang Un, from Chungnam National University, was the author of the most publications, with 67 articles. The study of hairy roots was more and more inclined to the study of gene and molecular function, including plant genetic transformation. Secondary metabolism was another important research direction. In addition, the potential of hairy roots in medicinal plants and environmental restoration has also attracted the attention of researchers, but it was essential to strengthen the cooperation among global hairy root researchers, deepen the research results and expand the depth of research directions.

## Data Availability

The original contributions presented in the study are included in the article/supplementary material. Further inquiries can be directed to the corresponding author.
